# Advances in Mitochondria-Targeted Drug Delivery

**DOI:** 10.3390/pharmaceutics15082089

**Published:** 2023-08-05

**Authors:** Emanuela Bottani, Dario Brunetti

**Affiliations:** 1 Department of Diagnostic and Public Health, Section of Pharmacology, University of Verona, 37134 Verona, Italy; 2 Unità di Genetica Medica e Neurogenetica, Fondazione IRCCS Istituto Neurologico Carlo Besta, 20126 Milan, Italy; 3 Department of Medical Biotechnology and Translational Medicine, University of Milan, 20129 Milan, Italy

Mitochondria are dynamic organelles that play a crucial role in numerous cellular activities. Their primary function is to convert the energy stored in nutrients into adenosine triphosphate (ATP) through the oxidative phosphorylation (OXPHOS) pathway. Mitochondria also play a pivotal role in other cellular functions, such as heme biosynthesis, oxygen sensing, calcium homeostasis, cell growth, fate, and death.

Whether primary or secondary, mitochondrial dysfunctions are increasingly recognized as a hallmark of several pathologies, including cancer, neurological, cardiovascular, immunological, and metabolic disorders; the central role of mitochondria in disease progression has made them an attractive target for therapeutic interventions. In this Special Issue, 88 authors from twelve countries have contributed with five original research articles and eight reviews, providing important insights into the field of mitochondrial dysfunction and mitochondrial-targeted therapeutic strategies to treat these conditions.

Mitochondrial dysfunction has been implicated in several diseases, including primary mitochondrial diseases (MDs), a group of severe genetic disorders caused by mutations in the nuclear or mitochondrial-genome-encoding proteins involved in the oxidative phosphorylation (OXPHOS) system. MDs have a wide range of symptoms, ranging from organ-specific to multisystemic dysfunctions, with different clinical outcomes. Currently, no effective therapies exist for these diseases [1,2]. The most immediate chance in the case of loss-of-function mutations, which characterised most primary mitochondrial diseases with nuclear genes affected, would be the exogenous re-expression of the functionally encoded protein. Additionally, promising evidence emerges from mitochondrially targeted, DNA-editing enzymes that effectively correct mutations in the circular genome of the organelle. Therefore, a growing number of pre-clinical and clinical trials over the last decade have shown that gene therapy is a viable precision medicine strategy for treating MDs, although impediments exist, including vector design, tropism, efficient delivery, transgene size and expression, and immunotoxicity. Last but not least, the astonishing costs of preclinical and clinical research to move gene therapy a step closer to the treatment of MDs halt the process at its very launch [3]. Di Donfrancesco et al. presented a comprehensive overview of the state-of-the-art of gene therapy in MDs, addressing the main challenges, the most feasible solutions, and the future perspectives of the field [4].

Mitochondrial dysfunctions play a pivotal role in several neurodegenerative diseases. Two original types of research included in this Special Issue were focused on Neurodegeneration with Brain Iron Accumulation (NBIA): a rare, inherited, neurological movement disorder characterized by extrapyramidal symptoms and by common pathognomonic evidence of accumulation of local iron in the brain with progressive degeneration of the nervous system [5]; mutations in different genes have been linked to NBIA [6,7,8,9]. Among these, pantothenate kinase-associated neurodegeneration (PKAN) is caused by a genetic alteration in *PANK2*, encoding for the mitochondrial enzyme pantothenate kinase-2, whose function is to catalyse the first reaction of the Coenzyme A (CoA) biosynthetic pathway, and for which no effective cure is available. In the first original research article, Santambrogio et al. [10] compared the pharmacological effects of leriglitazone, a novel brain-penetrant peroxisome proliferator-activated receptor gamma (PPARγ) agonist, to those of CoA on hiPS-derived astrocytes from three healthy individuals and three PKAN patients with different pathogenic mutations in *PANK2* gene. Leriglitazone (5-[[4-[2-[5-(1-hydroxyethyl)pyridin-2-yl]ethoxy] phenyl]methyl]-1,3rdiazolidine-2,4-dione hydrochloride), developed by Minoryx Therapeutics, is the hydrochloride salt of the active metabolite M4 (M-IV) of pioglitazone (Actos^®^, Takeda) that could modulate multiple biological pathways, including mitochondrial biogenesis and anti-oxidant defences [11], mitochondrial proteostasis [12], energy metabolism, and inflammation, which are relevant for neuroinflammatory and neurodegenerative diseases such as X-linked adrenoleukodystrophy (X-ALD) [13] and Friedreich’s ataxia (FRDA). Interestingly, this original work showed that the treatment with leriglitazone improved mitochondrial respiration, reduced iron accumulation, and increased PKAN-astrocytes’ vitality to a similar or even greater extent than CoA supplementation did, representing a promising drug to treat NBIA patients [10]. The proof that leriglitazone and CoA treatments have comparable effects suggests that the two compounds act on pathological mechanisms that share common alterations and allowed the authors to critically discuss and make a new hypothesis about the mystery of iron deposition in PKAN pathogenesis, which might be due to a non-use of iron in the dysfunctional mitochondria.

Mitochondrial membrane protein-associated neurodegeneration (MPAN) is a rare form of NBIA associated with autosomal recessive and dominant pathogenic variants in the gene C19orf12 [14], which encodes for a protein of an unknown function yet is associated with a potential role in lipid metabolism. In the study of Zanuttigh et al., the authors used MPAN fibroblasts from several patients with autosomal recessive and autosomal dominant forms to identify shared consistent phenotype(s) for testing pharmacological treatments. They provided convincing evidence that detailed that impaired autophagy is the only significant readout in MPAN fibroblasts, which can be used to screen small molecules with potential therapeutic effects in MPAN [15]. Indeed, five drugs acting as autophagy modulators able to increase the LC3 levels in patient fibroblasts (i.e., carbamazepine, LY294002, ABT-737, oridonin, and paroxetine) emerge as novel potential therapeutic compounds to treat MPAN [15].

Impaired mitophagy is also one of the hallmarks of the pathogenesis of Parkinson’s disease (PD), which highlights the importance of mitochondrial homeostasis and dynamics for the functioning of dopaminergic neurons. Not surprisingly, pathogenic mutations in Pink1 and Parkin proteins involved in the mitophagic process have been associated with the development of PD and to the accumulation of dysfunctional mitochondria in the neuron body. Blagow et al. critically analysed recent findings, advantages, and disadvantages of mitophagy activator compounds for the treatment of Parkinson’s disease, including the PINK1 and PARKIN activators and USP30 inhibitors [16].

In addition to PD and MDs, several diseases, including diabetes, heart failure and ischemia-reperfusion damage, cancer, amyotrophic lateral sclerosis (ALS), and Alzheimer’s disease, have been linked to mitochondrial dysfunctions, oxidative stress, and altered OXPHOS function. The identification of mitochondria as an emerging pharmaceutical target has led to the development of several mitochondrial targeting strategies for the treatment of these diseases. Khan et al. discussed the recent advancements in mitochondria-targeted drug delivery strategies, including small lipophilic cationic molecules, mitochondria-targeting signal peptides, penetrating peptides, and nanoparticle-based drug delivery, as well as their challenges and limitations [17].

Passing to the complex field of mitochondria and cancer, one original research article and a review contributed to this Special Issue. Li et al. presented a study centered on the role of celastrol, a potent triterpenoid, as a new anti-cancer drug with notable anti-angiogenic properties. This study shows that celastrol hampers tumour angiogenesis by suppressing mitochondrial function and altering morphology through the STAT3/OPA1/P65 pathway, thereby offering novel insights into mitochondrion-targeted cancer therapy [18].

One of the most effective chemotherapies, extensively used in the clinical settings of human cancer, is Doxorubicin (DOX), which, however, has the disadvantage of exerting cardiotoxicity, cardiomyopathy, and, eventually, heart failure. Alteration of the mitochondrial fission/fusion dynamic processes has been identified as a potential mechanism underlying DOX cardiotoxicity. DOX-induced excessive fission in conjunction with impaired fusion could severely promote mitochondrial fragmentation and cardiomyocyte death, compromising the clinical effectiveness of the chemotherapy. Maneechote et al. comprehensively revised the most recent in vitro and in vivo studies supporting the role of mitochondrial dynamics in DOX-induced cardiotoxicity and presented novel insights into the development of pharmacological interventions targeting mitochondrial fission/fusion to halt the cardiotoxic effects of DOX. This study encourages future clinical investigations to focus on the potential applications of mitochondrial dynamic modulators to counteract DOX-induced cardiotoxicity [19].

Due to their high energy demand, the heart, skeletal muscle, and kidneys are among the principal organs affected by mitochondrial dysfunctions. Huang et al. affronted this theme in a brilliant review discussing the alterations of mitochondrial metabolism in cardiovascular disease (CVD), the potential mitochondrial targets for CVD prevention and treatment, and the therapeutic strategies currently available at the preclinical and clinical levels [20]. Duchenne muscular dystrophy (DMD) is a progressive hereditary disease caused by the absence of the dystrophin protein, which is secondarily associated with a dysregulation of ion homeostasis, in which mitochondria play an important role. In their work, Dubinin et al. demonstrated that mitochondrial dysfunction in the skeletal muscles of dystrophin-deficient *mdx* mice is accompanied by a reduction in mitochondrial K^+^ transport and a decrease in K^+^ content in the matrix. This was associated with a decrease in the expression of the mitochondrial large-conductance, calcium-activated potassium channel (mitoBK_Ca_) in the muscles of *mdx* mice. Interestingly, the BK_Ca_ activator NS1619 normalised mitoBK_Ca_ expression and potassium homeostasis, increased the calcium retention capacity, mitigated the oxidative stress, and improved the mitochondrial ultrastructure, prospecting the use of this molecule in treatments of this secondary channelopathy [21].

The process of ageing is characterised by a general, gradual decline in cellular functions, which clinically manifests as the progressive loss of neuromuscular functions (i.e., frailty, cognitive decline and sarcopenia). The essential cellular and molecular characteristics of ageing are mitochondrial dysfunction and cellular senescence. This theme was well explored and reported in the review of Protasoni and Serrano, who delved into the primary changes observed in mitochondrial function during both ageing and cellular senescence, emphasizing the distinctions and similarities between these two processes. Additionally, they discussed the existing treatments that aim to intervene in these pathways and contemplate potential future directions for anti-ageing and anti-senescence therapies focusing on mitochondria [22]. In age-related sarcopenia, mitochondria homeostasis is disrupted because of reduced oxidative phosphorylation and ATP generation, the enhanced production of reactive oxygen species, and impaired antioxidant defence [23]. In their review, Bellanti et al. presented recent evidence on mitochondrial defects that are relevant in the pathogenesis of sarcopenia and that may represent promising therapeutic targets for its prevention/treatment. They also described muscle-mitochondria-targeted therapy for the management of sarcopenia, including the delivery systems, the most promising molecules, and their potentials to prevent sarcopenia in aged people [24].

The kidney has the second highest level of mitochondria and oxygen use after the heart, resulting in one of the most energy-necessitating organs in the human body. Mitochondrial dysfunctions—e.g., alterations of mitochondrial biogenesis, mitochondrial fusion–fission imbalance, mitochondrial-damage-associated molecular patterns, and oxidative stress, among others—have recently been linked to the pathophysiologies of various forms of kidney diseases and potentially provide alternative approaches to their treatments. Tarniover et al. reviewed the recent findings in the field of mitochondrial dysfunction and kidney disease and discussed the potential contributions of mitochondria-targeting drugs as a therapeutic approach [25].

Finally, mitochondrial dysfunctions can also be caused by environmental contaminants; an example was provided by Alvi et al. that explored the toxic effects of bisphenol A (BPA) in experimental mice. They found that BPA exposure induced oxidative stress, impairing lipid and amino acid metabolism, and demonstrated that these effects could be, in part, ameliorated by resveratrol treatment [26].

In conclusion, this Special Issue presents a set of diverse themes related to mitochondrial dysfunctions and mitochondrial-targeted drug delivery in several common and rare diseases. It is known that mitochondrial dysfunctions play an important role in the etiopathogenesis of many diseases, so we recognize that the range of topics included here is not in any way exhaustive. Nevertheless, we expect that this collection will be of value to all researchers interested in the pharmacological modulation of mitochondrial (dys)function in a wide range of clinical and preclinical settings.

This Special Issue would not have been possible without the massive work and insights of all the participating authors and reviewers, whom we sincerely thank for their efforts in making the articles here presented instructive to all interested audiences.
